# Interaction of *Salmonella* Typhimurium with Dendritic Cells Derived from Pluripotent Embryonic Stem Cells

**DOI:** 10.1371/journal.pone.0052232

**Published:** 2012-12-28

**Authors:** Raffaella Rossi, Christine Hale, David Goulding, Robert Andrews, Zarah Abdellah, Paul J. Fairchild, Gordon Dougan

**Affiliations:** 1 Wellcome Trust Sanger Institute, Wellcome Trust Genome Campus, Cambridge, United Kingdom; 2 University of Oxford, Sir William Dunn School of Pathology, Oxford, United Kingdom; Karolinska Institutet, Sweden

## Abstract

Using an *in vitro* differentiation protocol we isolated cells with the properties of dendritic cells (DCs) from immunologically refractive pluripotent murine embryonic stem cells (ESCs). These ES-derived dendritic cells (ESDCs) expressed cytokines and were able to present antigen to a T cell line. Infection of ESDCs with *Salmonella* Typhimurium stimulated the expression of immune cell markers and thousands of murine genes, many associated with the immune response. Consequently, this system provides a novel *in vitro* model, amenable to genetic modification, for monitoring host/pathogen interactions.

## Introduction

Dendritic cells (DCs) play a central role in the immune response, acting as sentinel cells for the detection and presentation of antigens to other immune cells. They are involved in the recognition and processing of antigens from potentially pathogenic microbes and are important for priming and stimulating immune cells involved in mediating protection against such agents. Conversely, invading microbes have developed mechanisms for subverting the activity of DCs. Some pathogens can interfere with the antigen presentation process or even stimulate innate death responses in DCs [1], [2]. Others, such as *Salmonella enterica*, actually exploit activated DCs as portals of entry into their hosts, using them to traverse the epithelial barriers of the intestine [3], [4].

*Salmonella* are potentially pathogenic for different vertebrate hosts, causing a variety of disease syndromes ranging from localized gastroenteritis to systemic typhoid [5]. One of the key pathogenic traits associated with the *Salmonella* is their ability to actively invade and persist within eukaryotic immune cells, including DCs [6], [7], [8]. These bacteria harbor specific sets of genes that facilitate the invasion and persistence processes and moderate vesicular trafficking within the targeted cells [9], [10]. There is evidence that they can also moderate the immune response by interfering with the cellular responses leading to both innate and acquired immunity [11], [12], [13].

Both animal studies, such as those based on the mouse, and *in vitro* models using cultured cells have proven valuable for studying host-pathogen interactions, including those involving *Salmonella*. Genetically manipulated mice and eukaryotic cells have added an extra dimension to the versatility of these approaches [14]. Cell based studies have normally relied on populations of primary cells grown directly from the host or on transformed and established differentiated cells adapted for tissue culture in the laboratory. Another potential approach is to exploit pluripotent embryonic stem cells (ESCs) as the host, as these can potentially be differentiated along different lineage pathways or genetically manipulated to create targeted and defined mutant lines [15]. Early studies on pluripotent ESCs from mice have shown them to lack immunological competence. For example, murine ESCs exposed to different pathogenic bacteria, including *Salmonella enterica* serovar Typhimurium (*S*. Typhimurium), have no obvious innate or other immune signature even though they are readily invaded by these bacteria [16]. Protocols have been developed for reproducibly differentiating pluripotent ESCs into immune cells, including those of the DC lineage [17]. These protocols enable the potential to study the immune response stimulated by pathogens in carefully controlled cellular environments, including the application of genetic modification. Here we report methods for the exploitation of ESC derived DCs for the investigation of the immune response to *Salmonella*. These approaches are potentially applicable to a range of pathogens and their products.

## Materials and Methods

### Mouse Cell Culture and ESC Differentiation into DCs

Murine ESC line AB2.2 (129/Sv/EvBRD-Hprtb-m2) [18] was a gift of Professor Allan Bradley [19]–[20]. They were induced to differentiate into DCs following the published protocol [17]. Briefly, AB2.2 mouse ESC line at low passage number was grown in Knockout Dulbecco’s Modified Eagle’s Medium (Knockout DMEM, Gibco), 15% Fetal Bovine Serum (FBS) (Hyclone), 2 mM L-Glutamine, 2 mM 2-Mercaptoethanol stock solution. After thawing, the cells were grown on irradiated STO’s/SNL 76/7 embryonic feeder cells producing Leucocyte Inhibitory factor (LIF) and then for two passages in flasks coated with 0.1% gelatin in feeder-free-media containing 500–1000 U/ml LIF. The ESCs were then seeded at 4×10^5^ cells/9 cm Petri dish with 20 ml LIF-free media to form embryoid bodies. Ten-fourteen day old embryoid bodies were seeded in 20 ml of medium containing 25 ng/ml Granulocyte-Macrophage Colony Stimulating Factor (GM-CSF) and 200 WHOSU/ml rmIL-3 in culture dishes. The differentiation assay was performed multiple times for optimization using independently frozen ESCs batch. Specifically all Flow Cytometric and antigen presentation assays were performed at least three times. T cell line MF2.2D9 [21] was maintained in DMEM media enriched with 10% FBS, 2 mM L-Glutamine, 1 mM Sodium Pyruvate, 1× MEM-NEAA, 1∶1000 of 2-ME stock solution, penicillin and streptomycin 1∶1000.

### Bone Marrow DCs (BMDCs)

Mice were sacrificed by neck dislocation. Bone marrow from the tibia and femur of 5–10 week old 129/Sv mice were flushed using a syringe and needle containing RPMI-1640, 1× penicillin/streptomycin and 2% FBS. The cells were allowed to settle for 5 min at room temperature, the supernatant was transferred in a fresh tube and bone marrow collected by centrifugation. The cells were suspended and cultured in Iscoves modified Dulbecco’s medium (IMDM) with 10% FBS, 2 mM L-Glutamine, 1 mM Sodium Pyruvate, 1× MEM-NEAA, 2-β-Mercaptoethanol and penicillin/streptomycin. The medium was supplemented with differentiation factors GM-CSF at 500 WHOSU/ml and IL-4 at 150 WHOSU/ml. The bone marrow derived DCs were harvested at 6 and 9 days for analysis. The procedure was ethically approved by the Wellcome Trust Sanger Institute’s Ethical Review Committee and all animal work was carried out in accordance with UK Home office regulations under licence number 80/2099.

### Flow Cytometric Analysis of Surface Cell Markers

The mouse ESC line AB2.2 and the embryonic stem cell derived DCs were washed with PBS, incubated with cell dissociation buffer (Sigma), centrifuged and fixed in 1% paraformaldehyde in PBS for 20 minutes at 4°C, washed with PBS and incubated with specific antibodies including CD44 PE-Cy5-conjugated (BD Biosciences); Integrin α-6 (CD49f, R&D Systems) and CD11b APC-conjugated (BD Biosciences); Octamer-Binding Protein 3/4 (OCT3/4, R&D Systems), Stage-Specific Mouse Embryonic Antigen 1 (SSEA-1), CD11c and CD80 PE-conjugated; CD86, IA/IE and H-2K FITC-conjugated (all from BD Biosciences). Cellular membranes were permeabilized with saponin buffer for intracellular staining when needed. Similar isotype matched antibodies conjugated to the same fluorophore were employed as negative controls (BD Biosciences). Surface marker expression analysis was performed using a FACS Aria I (BD Biosciences).

### Bacterial Growth and ES Cell Infection

*S.* Typhimurium SL1344(p1C/1), which expresses GFP under the control of the promoter *ssaG* [22], were used to infect cells. In order to carry out *in vitro* infection, *S.* Typhimurium SL1344(p1C/1) were grown from a single colony in Luria Bertani broth with 50 µg/ml Ampicillin selection at 37°C over night in static conditions until OD_600_ ∼ 0.6–0.7. The bacterial suspension was diluted with culture medium to a desired multiplicity of infection (MOI) of 100 and added to the culture cells. The *in vitro* cells were incubated with bacteria for 30 minutes at 37°C and after 2 washes with PBS they were incubated with culture media containing Gentamicin at 50 µg/ml for the indicated time.

### Confocal Microscopy

To track bacterial intra-cellular location, ESC derived DCs were grown on glass coverslips treated with poly-L-lysine and infected with *S.* Typhimurium SL1344(p1C/1). At the indicated time, the cells were washed and fixed with 1% paraformaldehyde buffer. They were then permeabilized with saponin buffer and stained with primary antibodies against Early Endosome Marker 1 (EEA-1) (Rabbit polyclonal primary antibody, AbCam), Lysosome Associated Membrane Protein 1 (LAMP-1) (Rat monoclonal primary antibody, AbCam) or LAMP-2 (Rat monoclonal primary antibody, AbCam) respectively following the manufacturer’s recommended dilutions, incubated for 30–40 minutes, prior to being washed. Finally they were incubated in the dark with secondary goat anti-rabbit or goat anti-rat antibodies conjugated to APC-Cy7 (Santa Cruz Biotechnology**)** according to the manufacturer’s instructions. After washing, the coverslips were mounted on glass slides with ProLong Gold containing DAPI (Invitrogen).

### Electron Microscopy

Cells were fixed in culture medium containing 2.5% glutaraldehyde and 4% paraformaldehyde at 37°C for 5 minutes and then transferred to ice for 25 minutes. Cells were then scraped from the dish with a freshly cut Teflon strip and transferred to an Eppendorf centrifuge in fresh cold fix, pelleted and fixed for a further 30 minutes. The pellet was carefully rinsed in 0.1 M cacodylate buffer and post-fixed for one hour at room temperature in 1% buffered osmium tetroxide followed by a brief rinse and then mordanted for one hour in 1% buffered tannic acid, briefly rinsed in 1% aqueous sodium sulphate and dehydrated in an ethanol series with 2% uranyl acetate added at the 30% stage. Finally the cell pellets were infiltrated and embedded in TAAB 812 resin at 60°C for 24 hours. Ultrathin sections were cut on a Leica UCT ultramicrotome, contrasted with uranyl acetate and lead citrate and imaged on an FEI 120 kV Spirit Biotwin with a Tietz F415 CCD camera.

### Cell Maturation and Cytokine Measurement

Bone marrow and ESC derived DCs were incubated overnight with the activating agents *S.* Typhimurium LPS (Sigma) at 10 µg/ml or Tumour Necrosis Factor α (TNFα) (R&D Systems) at 5000 U/ml and cytokine measurements were carried out on the culture supernatants. These cells were also used for confocal imaging. Undifferentiated ESCs and ESDCs were harvested and incubated overnight then exposed to *S.* Typhimurium for 2 and 4 hours at a multiplicity of infection or MOI 100 and their ability to secrete cytokines into the supernatant was tested. Cytokine concentrations were measured using Mouse Cytometric Bead Array (CBA) kit from BD Biosciences that detects and measures the concentration of the cytokines Interleukin 6 (IL-6), IL-10, IL-12, TNFα, Monocyte Chemotactic Protein-1 (MCP-1) and Interferon γ (IFNγ) captured onto beads. The experiment was performed following the manufacturer’s instruction. The cytokine bound beads were washed once in PBS and analyzed with a FACS Aria I. The data obtained was processed with FCAP Array software. T cell activation was measured by the production of IL-2 upon antigen presentation using mouse CBA IL-2 Flex Kit (BD Biosciences).

### Antigen Presentation Assay

Allogeneic T cell hybridoma MF2.2D9 (specific for I-A^b+^OVA258-276 peptide Major Histocompatibility Complex (MHC) class II restricted peptide, gift from Dr B. Chain, University College London) were seeded with DCs at a 5 to 1 ratio in 96 well flat bottom plates (in triplicate). Whole ovalbumin protein was added at 10 µg/ml. As a positive control MF2.2D9 T cells were supplemented with 5 µg/ml of Concanavalin A (ConA). All cells were incubated for 24 hours at 37°C, 5% CO_2_ and the supernatants were obtained without disturbing the cells and stored at −20°C. Levels of IL-2 expressed by activated T cells were measured by using a mouse CBA IL-2 Flex Kit assay (BD Biosciences).

### Cell Sorting and Total RNA Extraction

Infected and uninfected ESC derived DCs were sorted by FACS (Aria cell sorter I, BD Biosciences). Single cell suspensions were obtained using cell dissociation buffer and the cells were maintained in PBS during sorting. The cells were sorted into sterile 15 ml Falcon tubes containing 2 ml of RNA*later*® solution (Ambion). Total RNA was extracted using a RNeasy Mini kit (QIAGEN). Additionally, an uninfected sample of cells was also sorted to obtain total RNA from uninfected cells. The total RNA quantity was measured by Spectrophotometer NanoDrop-1000 v 3.1.0 (Thermo Scientific) and the RNA quality was analyzed using a 2100 Bioanalyzer (Agilent) following the manufacturer’s instructions. The differentiation protocol was performed at least three times in order to carry out infection and cell sorting for total RNA extraction.

### Microarray Hybridization and Data Analysis

Microarray chip hybridization was carried out following the manufacturer’s instructions. Briefly, Biotin-labeled cRNA was synthesized from 300 ng total RNA with the TotalPrep RNA Amplification kit (Ambion, Foster City, CA, USA). The hybridization mix containing 1500 ng of labeled cRNA was prepared according to the Illumina BeadStation 500× System Manual (Illumina, San Diego, CA, USA). The hybridization of Illumina MouseWG-6 v1.1 Expression BeadChip array was performed overnight at 58°C on the BeadChip Hyb Wheel. The BeadChips were washed with E1BC solution for 15 minutes, and then washed in 100% Ethanol for 10 minutes and after a final wash in E1BC for 2 minutes they were treated with Block Buffer E1 on a rocker mixer for 10 minutes. The staining reaction was performed using 2 ml of buffer E1 with 1 µg/ml of Cy3-streptavidin followed by a 5 minutes’ wash with buffer E1BC. The BeadChips were then dried by centrifugation and scanned using the Illumina BeadStation 500 platform operated by Illumina BeadArray Reader software. For each time point, three independent sample replicates were hybridized to three different chips.

Microarray data were analyzed using Bioconductor [23] to perform a pair-comparison analysis between mRNA expressed by uninfected cells versus infected cells at 2 hours and 4 hours post infection. The data obtained from Bioconductor analysis were further analyzed for cellular Pathways enrichment using InnateBD database [24]. The corresponding human orthologs were determined and the results were enriched using the ‘over-representation analysis’ option which allows selection of genes on the basis of their fold change (+/−1.5) and p-value (0.05). The default settings for the analysis algorithm (Hypergeometric) and the multiple testing correction method (The Benjamin & Hochberg correction for the False Discovery Rate (FDR)) were used. Only pathways that are significantly up- or down- regulated with p-value <0.05 are reported here.

### Semi-quantitative Real Time-PCR

The cDNA was synthesized using QuantiTect Rev. Transcription kit (Qiagen) from 30 ng of total RNA following the manufacturer’s instructions. Primers were designed using primer3 website [http://frodo.wi.mit.edu/primer3]. Primers are shown in Table 1. The RT-PCR reactions were performed using SYBR green dye from SensiMix Plus SYBR kit (Quantace) on a Mx3000P RT-PCR machine (Stratagene).

**Table 1:** Primers for semi-quantitative Real Time-PCR.

Gene Symbol	Ensembl Number	Forward primer 5′-3′	Reverse primer 5′-3′
S18	ENSMUST00000024763	GAACACCGAAAAATCGAGGA	CGGTTGAGCTTGGGTTTATC
Tyki/Cmpk2	ENSMUST00000020969	ATCTCGTGGCTTCTGAAATAGC	ACCTCAGTAGCTATGGCGTAGG
Oasl1	ENSMUSG00000041827	AGCGAAACTTCGTGAAGCA	GCTTCCCAGGCATAGACAGT
Cxcr4	ENSMUSG00000045382	ACGGCTGTAGAGCGAGTGTT	AGGGTTCCTTGTTGGAGTCA
H2-DMa	ENSMUSG00000037649	TGACAAAAGCTTCTGCGAGAT	GCTGATGAAACAGACCAACG
Lypla3/PLA2G15	ENSMUST00000034377	TGGCCTCCTGTTACCTCTGT	GTCCGTCTTCTTGGAGCAAA
IL6	ENSMUSG00000025746	GAGCCCACCAAGAACGATAG	GTGGTTGTCACCAGCATCAG
H2-T9	ENSMUSG00000056116	ACAGCTGTCTGAAAGGAATCTG	CTCCACATCGCAGCCTTG

Each sample was run in triplicate for each biological replicate. To test the primers for specificity and quality, efficiency curves were performed and dissociation products were obtained. The Ct (threshold cycle) values were analyzed for relative quantification using ribosomal protein S18 transcript as housekeeping control gene. The results are reported as Ln2∧−(ΔΔCt).

### Statistics

Colony formation units (CFUs) were reported as mean of three counts +/−1 standard deviation (SD). Cytokine concentrations were analyzed using one-way ANOVA test. Semi quantitative real time RT-PR data were analyzed using REST© software [25].

### Microarray Accession Number

Microarray data are published in ArrayExpress experiment number E-MTAB-1392.

## Results

### Differentiation and Characterization of DCs from ESCs

In a previous study we characterized the interactions between undifferentiated pluripotent mouse ESCs and a number of pathogenic bacteria, including *S.* Typhimurium and *Shigella flexneri* [16]. Interestingly, although *S.* Typhimurium was able to invade and persist within such ESCs there was virtually no evidence for the activation of genes associated with innate or adaptive immune responses. This observation encouraged us to investigate if ESCs could be differentiated into a cell type that can mount a pronounced immune response following exposure to this pathogen. DCs play a key role in the pathogenesis of salmonellosis and the subsequent host immune response. Thus, we explored these interactions following differentiation of ES cells along the DC lineage [26].

**Figure 1:**
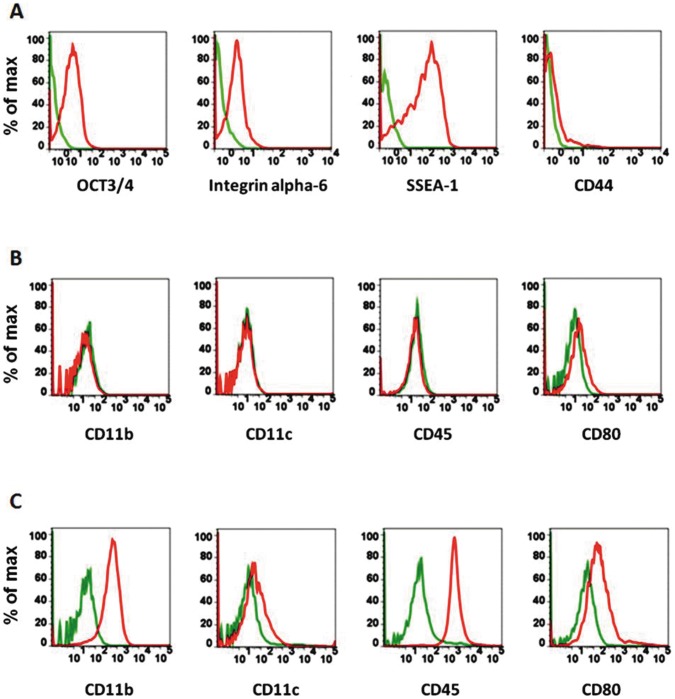
The nature of un-differentiated or differentiated cells was tested by flow cytometry. Flow cytometry on ESCs and ESDCs was used to monitor the expression of different cell surface markers. (A) Stem cell pluripotency markers on undifferentiated ESCs and (B) Dendritic cell markers on undifferentiated ESCs confirm their pluripotency and self-renewal nature; (C) Dendritic cell markers expressed on ESDCs confirm the change in APC. Green lines represent cells stained with control isotype, red lines are cells stained with the relevant antibody.

**Figure 2:**
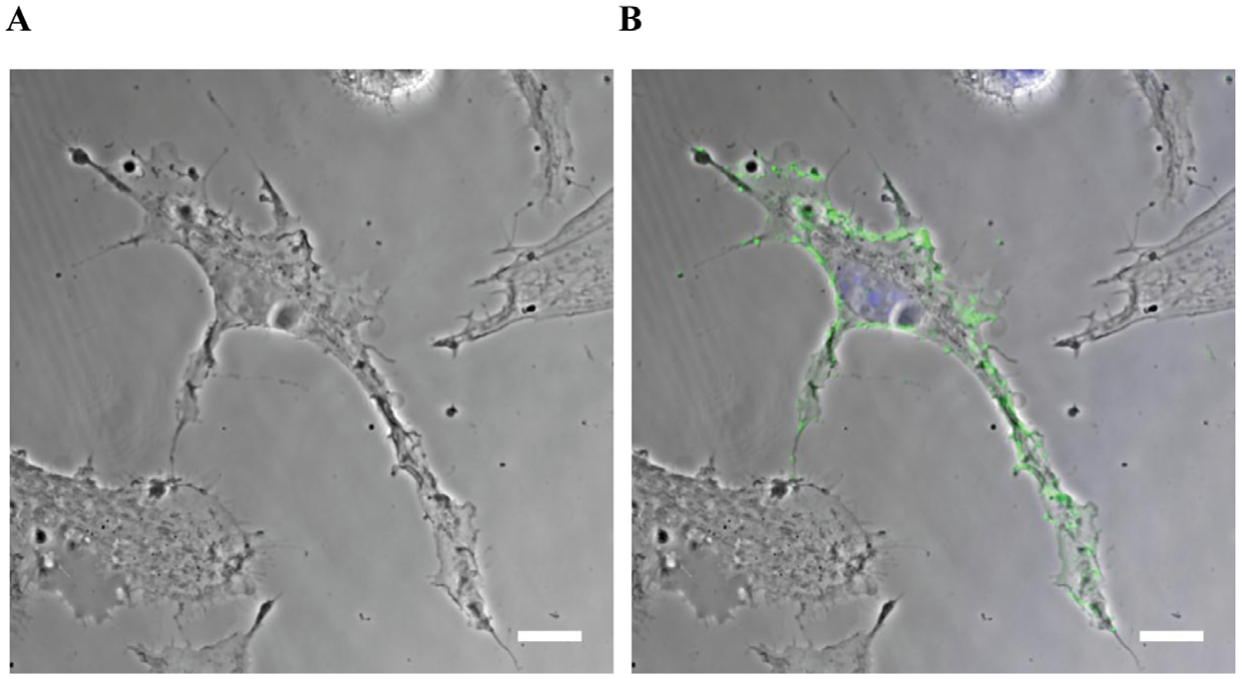
Morphology of ESDC and MHC-II expression was observed by confocal microscopy. Stimulated ESDCs were imaged using a Confocal Zeiss LSM510; (A), phase contrast, (B) cell stimulated with TNFα and stained with anti-MHC Class II FITC conjugated antibody. Scale bar 10 µm.

Undifferentiated mouse ES cells derived from the line AB2.2 were initially characterized for surface marker expression by flow cytometry. Such cells expressed markers characteristic of pluripotent and self-renewing ESCs including Oct3/4, Integrin α-6 and SSEA-1 whilst being negative for CD44, a marker used to confirm the undifferentiated status of pluripotent and self renewing cells (Fig. 1A). These cells were also analyzed for the expression of other markers normally associated with populations of leukocytes including CD11b, CD11c, CD45, CD80 (Fig. 1B) CD4, CD8, CD86, H-2K and IA-IE (Table 2). They were found to be generally negative for all these markers, although low levels of CD40 and CD54 were detected on some cells in the population. Next, to drive differentiation down the DC lineage, single ESCs were seeded under conditions lacking LIF for at least 10 days, the time necessary for the development of embryoid bodies. Subsequently these embryoid bodies were incubated with GM-CSF and IL-3 as described previously to drive them along the DC lineage. After about 4 days of incubation in the conditioned media, cells resembling DCs started to differentiate from the edges of the adherent embryoid bodies. Under the light microscope the ESC derived DCs were visible as bright cells growing in small clusters budding all around the culture dish. They were recognizable by characteristic comma-like protrusions. Resting dendritic-like cells were visible around both beating and non-beating embryoid bodies. Observations made using confocal and electron microscopy revealed striking morphology differences between undifferentiated ESCs and the corresponding DCs. Undifferentiated ESCs grew attached to a layer of gelatin, close to each other and often one on the top of the other. Once differentiated into DCs, they were visible via the microscope either as single cells or organized in small clusters. ES derived DCs had long and thin protrusions branching from the cell body, sometimes as dendritic extensions and other times as obvious lamellipodia (Fig. 2A).

**Table 2:** Flow cytometry analysis summary for surface markers expressed on different cell populations including AB2.2 murine ESCs, ESDCs no-stimulated or incubated with LPS or TNFα and no-stimulated BMDCs.

Marker	AB2.2	ESDCs			BMDCs	
		ESDCs	LPS 24 h	TNFα 24 h	BMDCs	LPS 24 h
CD11b	−	++	+++	+++	++	++
CD11c	−	++	+++	+++	++	++
CD4	−	+	++	+	N/A	N/A
CD40	+	++	+++	++	++	++
CD45	−	+++	+++	+++	++	++
CD54	+	++	+++	++	+	+
CD8	−	−	−/+	−	N/A	N/A
CD80	−	++	++	++	−/+	+
CD86	−	−	−/+	−	−/+	+
H-2K	−	+	++	+	++	+
IA/IE	−	−	−/+	−/+	+	++
TLR2	−	++	++	++	+	+
TLR4	−	+	++	+	+	−/+
TLR5	−	+	+	−/+	−/+	−
TLR9	−	+/−	+	−	N/A	N/A

+/− indicates ∼ 20% of the cells were positive;+indicates that at least 30% of the cells were positive;++indicates ≥60% of the cells were positive;+++indicates that ≥80% of the cells were positive for the marker tested.

**Figure 3:**
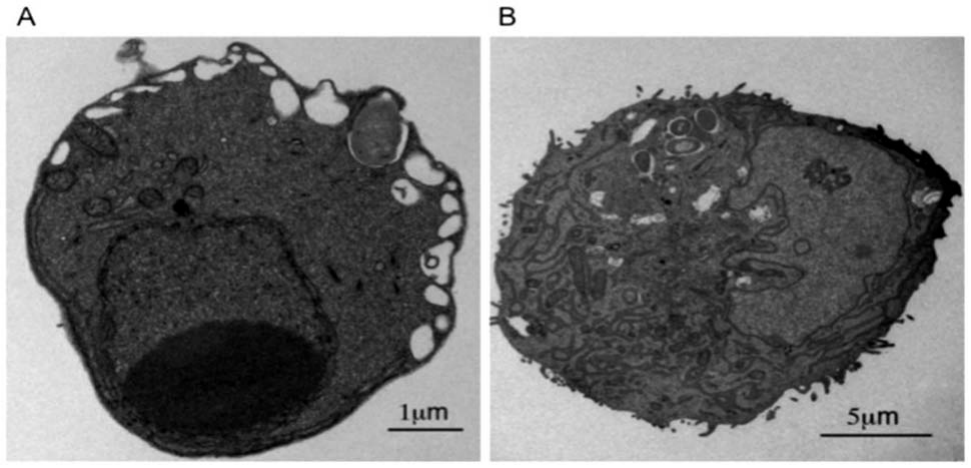
Electron microscope observations revealed details of internal organelles of undifferentiated and differentiated cells. Electron micrographs showing (A) undifferentiated ESC and (B) ESDC. Note the presence of intracellular *S.* Typhimurium in these images. Scale bars denote different sizes.

There was a striking difference in the organelles present in undifferentiated ESCs compared to the ES derived DCs when they were observed using electron microscopy (Fig. 3A and B respectively). The much larger ESDC (Fig. 3B) had a different nuclear/cytoplasm volume ratio and exhibited relatively increased numbers of cellular organelles including mitochondria and Golgi. In addition, features resembling MHC class II associated multi-laminar compartmental structures were observed in ESDCs but not in undifferentiated ESCs. Similar compartments were described previously in the DC line CB1 [12]. These data provide a visual confirmation that ES cells exhibit a different overall morphology after differentiation.

Populations of ESC derived DCs were harvested by vigorous pipetting and the detached cells were characterized for the expression of selected surface markers by flow cytometry. The ESC derived DCs had enhanced expression of multiple surface markers including CD11b, CD11c, CD45, CD80 (Fig. 1C), CD4, CD40, CD54, CD80, H-2K and selected Toll like receptors (TLRs) (Table 2) compared to undifferentiated ES cells (Fig. 1B and Table 2). Interestingly, these cells did not express detectably higher levels of some markers typical of activated classical DCs such as IA-IE and CD86.

Next, harvested ESDCs cells were incubated with either Tumor Necrosis Factor α (TNFα) or lipopolysaccharide (LPS) [27]. After overnight incubation with LPS, the ESDCs exhibited increased expression levels of some markers including H-2K CD11c, CD11b, CD4, CD40, CD54, TLR4 and MHC Class II (Table 2, Fig. 2B), compared to untreated ESDCs. Following treatment with TNFα, these cells exhibited increased expression of similar markers (Table 2). For comparative purposes flow cytometry was performed on murine BMDCs prepared as described in Methods. These BMDCs expressed all the markers used (Table 2).

### Antigen Presentation to T cells

A key characteristic of DCs is their ability to process and present specific antigens to naïve T cells using MHC Class II. Consequently, ESDCs were tested for their ability to process and present antigens by co-incubating them with the allogeneic naïve T cells line MF2.2D9 (I-A^b+^ OVA258-276 peptide-Class II specific T cell hybridoma) with or without whole ovalbumin. The cell line MF2.2D9 can respond to exposure to ovalbumin antigen presented on MHC class II by producing IL-2. The concentration of IL-2 in the culture supernatants was thus assessed after 24 hours of incubation with ESDCs and ovalbumin. BMDCs were used as positive controls. The level of IL-2 produced by the T cells mixed with either the ES cell derived dendritic cells or BMDCs was quantified by bead assay (Table 3, representative of independent experiments). Prolonged time incubation to 48 hours did not significantly increase the concentration of IL-2 (data not show). These experiments confirmed the ability of ESC derived DCs to specifically activate antigen specific T cells line.

**Table 3:** Concentration of IL-2 in the culture supernatant of naïve MF2.2d9 T cells mixed with either BMDCs or ESDCs incubated for 24 with ovalbumin, TNFα or alone with Concavallin A (ConA).

Cytokine pg/ml	BMDC	BMDC+T cells	BMDC+T cells+Ova at 24 h	BMDC+T cells + TNFα at 24 h	T cells+ConA
IL-2	0±0	8±4	87±135	3	437±290
**Cytokine pg/ml**	**ESDC**	**ESDC+T cells**	**ESDC+T cells+Ova at 24 h**	**ESDC+T cells + TNFα at 24 h**	**T cells+ConA**
IL-2	0±0	5.5±3.5	65±22	5	854±753

These data are representative of at least 2 independent experiments. Statistical analysis performed with one-way ANOVA.

Ovalbumin 10 µg/ml, TNFα 5000 WHOSU/ml, Concavallin A 5 µM/ml.

### Interaction of ESDCs with *S.* Typhimurium SL1344(p1C/1)

**Figure 4:**
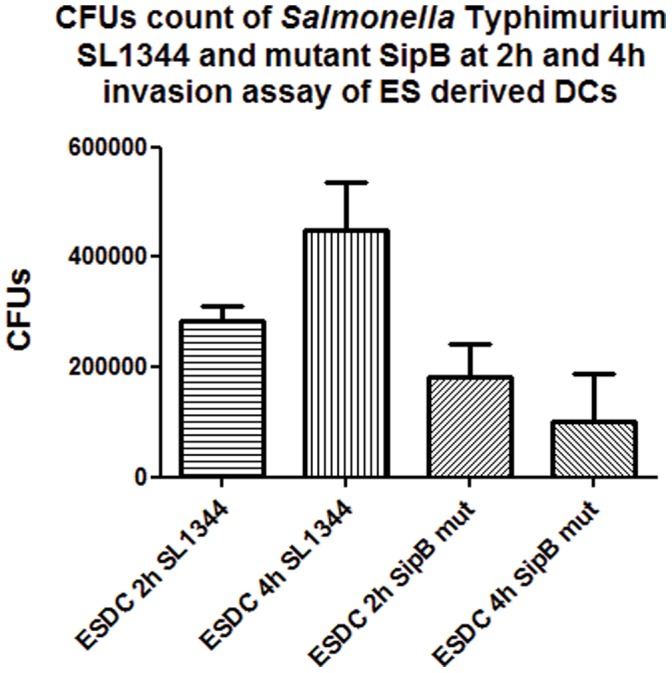
Gentamicin invasion assay. The ability of *S.* Typhimurium SL1344(p1C/1) to invade ESDCs was measured by the gentamicin assay described in Methods. ESDCs were exposed to either wild type *S*. Typhimurium or a *SipB* mutant for 30 minutes and then incubated with media containing 50 µg/ml of Gentamicin for 2 or 4 hours and then invading bacteria were enumerated.

**Figure 5:**
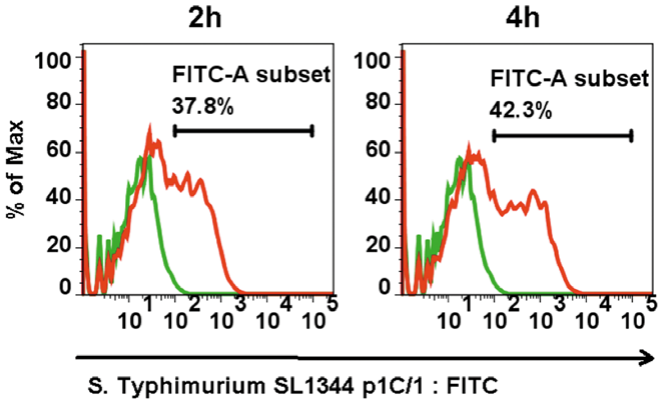
The invasion assay was also evaluated by Flow Cytometry. The ability of *S.* Typhimurium SL1344(p1C/1), able to express GFP once inside the host cell, to invade ESDCs was measured by monitoring the GFP expression using FACS analysis. Cells were analyzed for GFP expressing bacteria at time 2 and 4 hours post-infection. The horizontal bar in each histogram represents the percentage of fluorescent positive cells on 10.000 events. FITC subset in non infected cells is <1%. These data are representative of at least 3 independent experiments.

The ability of *S.* Typhimurium SL1344(p1C/1) to invade ESC derived DCs was investigated using a combination of microbiological counts, FACS and microscopy. Microbiological count of *S.* Typhimurium SL1344(p1C/1) was carried out using an optimized multiplicity of infection (MOI) of 100 bacteria per cell in each well. *S.* Typhimurium(p1C/1) *sipB*, a non-invasive mutant, was used as a control. Fig. 4 shows the colony forming units (CFUs) recovered at 2 and 4 hours post infection in this *in vitro* assay, following Gentamicin selection for intracellular bacteria. The number of bacteria recovered increased between 2 hours and 4 hours post infection but was lower after 24 hours of incubation (data not shown), due to increasing cell death of ES cell derived DCs [28]. FACS was carried out using *S.* Typhimurium SL1344(p1C/1), which harbors an *in vivo* activated promoter that drives the expression of Green Fluorescent Protein (GFP) once inside the host cell (Fig. 5). These data showed that more than 30% of ESDCs consistently harbored intracellular bacteria at 2 hours post-infection and between 35 and 42% at 4 hours post-infection.

**Figure 6:**
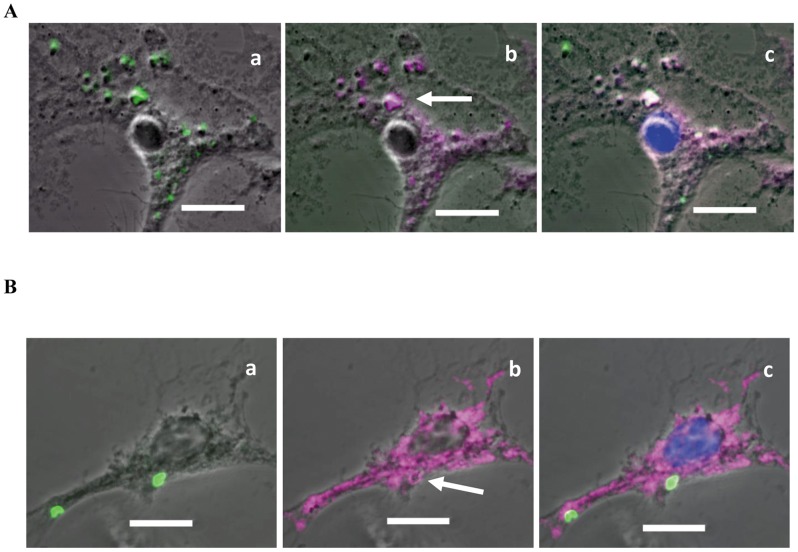
The interaction of *S.* Typhimurium with ESDCs was followed by fluorescent labeling. Confocal microscope images of ESDCs (phase contrast) infected with *S*. Typhimurium SL1344(p1C/1) expressing GFP, (A) co-labeled with early endosome marker EEA-1 at 30 minutes post-infection, (B) late lysosome marker LAMP-1 at 3 hours post-infection. Images are subdivided into (a) bacteria alone (FITC), (b) endosome compartment alone (APC-Cy7), with labeling arrowed. (c) Combined channels with DAPI added. Scale bar 10 µm.

ESDCs were tightly adherent to the plastic after exposure to *S.* Typhimurium SL1344(p1C/1). As early as 30 minutes after infection *S.* Typhimurium SL1344(p1C/1) were found co-localized with the EEA1 (Fig. 6.A) as has been previously described in BMDCs [29]. In addition at 3 hours post-infection the bacteria were often found co-localized with the late lysosome marker LAMP1 (Fig. 6.B).

### ESDCs Express Cytokines before and during *Salmonella* Infection

One important characteristic of DCs is the ability to produce cytokines and present antigens to other immune cells. For this reason we monitored the ability of cultured ESDCs to produce a number of cytokines including TNFα, IFNγ, IL-6, IL-12 subunit 70 (IL-12p70), MCP-1 and IL-10 in response to exposure to *S*. Typhimurium SL1344(p1C/1). Once differentiated into ESDCs the supernatants were sampled at 24 hours after harvesting and at 30 minutes, 2 hours and 4 hours after *S*. Typhimurium SL1344(p1C/1) infection. Low levels of MCP-1 and TNFα were already present in the culture supernatant of uninfected ES cell derived DCs (Table 4). After 30 minutes of *S.* Typhimurium SL1344(p1C/1) infection the supernatants from ESC derived DCs harbored IL-6, MCP-1 and TNFα. Statistical analysis showed a significant difference in the ESDCs expression at all infection time points for MCP-1 and at 4 hours infection of IL-6 and TNFα compared to undifferentiated ESCs. At 2 and 4 hours post infection, detectable levels of IL-10 were also present but this was not statistically significant. However, little or no IFNγ or IL-12p70 was detectable at any time during the *S.* Typhimurium SL1344(p1C/1) infection of the ESC derived DCs (Table 4). It is striking to note that no detectable levels of cytokines were produced by undifferentiated ESC during the time points here reported (Table 4 and reference 17).

**Table 4:** Concentration of cytokine produced by AB2.2 mouse ESCs or ESDCs, alone and during infection with *S.* Typhimurium SL1344(p1C/1).

Cytokines pg/ml	ESCs				ESDCs			
	0 min	30 min	2 h	4 h	0 min	30 min	2 h	4 h
IFNγ	0	0	0	0	0	0	0	0
IL-10	0	0	0	0	0	0	33±21	56±35
IL-12p70	0	0	0	0	0	0	0	0
IL-6	0	0	0	0	0	5.5±1.4	27±4	322±124*
MCP-1	0	0	0	0	203±21*	193±25 *	149±22*	282±56*
TNFa	0	0	0	0	45±5	177±24	1402±558	2984±1808*

Each value represents the average of at least two measurements ± average deviation. The detection limits of the assay were 2.5 pg/ml for IFNγ, 17.5 pg/ml for IL-10, 10.7 pg/ml for IL-12p70, 5 pg/ml for IL-6, 52.5 pg/ml for MCP-1 and 7.3 pg/ml for TNFα values below these are listed as 0 in the table).The data was analyzed using one-way ANOVA,

* indicates a significant difference with p<0.05.

### The Transcriptome of Murine ESDCs Infected by *S.* Typhimurium SL1344(p1C/1)

**Figure 7:**
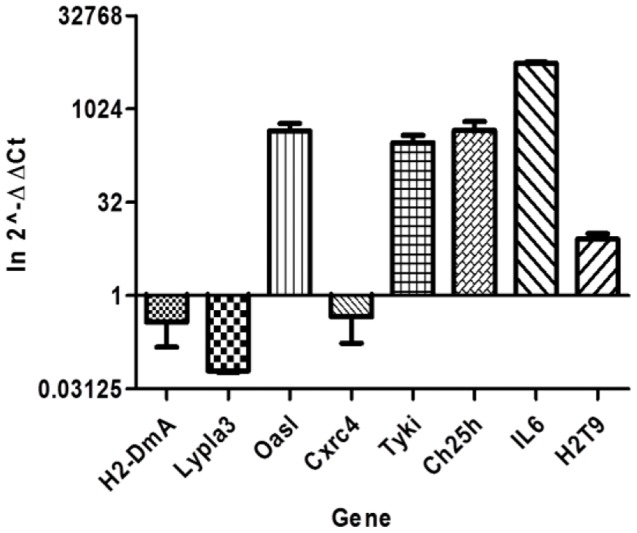
Graphic representation of the quantified expression of chosen genes versus un-infected ESDCs. Total RNA extracted from infected ESDCs was used in semi-quantitative Real Time-PCR to confirm the BioConductor analysis of up- or down-regulated genes. The data were analyzed using the ΔΔct value calculated versus the un-infected ESDCs.

Once we had established that ESDCs have the ability to present antigen, their RNA expression profile was investigated during *S.* Typhimurium SL1344(p1C/1) infection (Table 5 and 6). *Salmonella* infected cells were isolated from uninfected ESDC by fluorescent activated cell sorting into RNA*later* at 2 hours and 4 hours post-infection by exploiting the GFP tag associated with *S.* Typhimurium SL1344(p1C/1). The mRNA was extracted and cDNA was synthesized and hybridized on Illumina arrays carrying 35,000 mouse probes and the data were analyzed by pair-comparison using Bioconductor. This experimental analysis highlighted the ability of the differentiated cells to mount a strong response to bacterial invasion with 3615 genes significantly differentially expressed with p<0.01 at 2 hours post-infection. Of these genes, 325 had a >2 fold difference in expression levels and confidence p value <0.0001, compared to uninfected cells. One hundred genes showed a >4 fold differential expression with p-value <0.01 (one of which was down regulated). At 4 hours post-infection more than 4000 genes were significantly differentially expressed with p<0.01. Of these genes, 525 had an expression difference >2 fold compared to uninfected cells with a confidence p value of <0.001. One-hundred and 75 genes showed a >4 fold difference in expression with p-value <0.01. The Bioconductor analysis results were confirmed by semi-quantitative Real Time-polymerase chain reaction (PCR) performed on selected genes including those encoding IL-6, H2-Dma and Cxcr4 and these data were in agreement with the microarray analysis (Fig. 7). These included Oasl1 (2'-5' oligoadenylate synthetase-like 1) a gene involved in the innate immune response, Tyki (cytidine monophosphate (UMP-CMP) kinase 2, mitochondrial) an LPS-inducible member of the thymidylate kinase family and IL-6, which is produced by activated DC in vivo [30] and plays a role in DC maturation [31].

**Table 5:** Innate DB pathways up-regulated by ESC derived DCs at 4 hours post-infection.

GO term name of pathways up-regulated at 4 hours infection	Source Name	GO term up-reg./uploaded genes	GO term up-reg. p-value (corrected)
Cytokine-cytokine receptor interaction	KEGG	26/104	0.00049
IL4-mediated signaling events	PID NCI	12/33	0.00427
IL23-mediated signaling events	PID NCI	10/24	0.00444
Jak-STAT signaling pathway	KEGG	20/80	0.00583
CDK-mediated phosphorylation and removal of Cdc6^1^	REACTOME	11/36	0.01901
IL12-mediated signaling events	PID NCI	11/36	0.01901
Ubiquitin-dependent degradation of Cyclin D1^1^	REACTOME	11/36	0.01901
Ubiquitin Mediated Degradation of Phosphorylated Cdc25A^2^	REACTOME	11/35	0.02041
Vif-mediated degradation of APOBEC3G^3^	REACTOME	11/35	0.02041
Vpu mediated degradation of CD4^3^	REACTOME	11/34	0.02121
Gene expression of SOCS by STAT dimer (IFNγ signaling)	INOH	6/12	0.02833
Beta-catenin phosphorylation cascade^4^	REACTOME	11/39	0.03217
Degradation of beta-catenin by the destruction complex^4^	REACTOME	11/39	0.03217
CDT1 association with the CDC6:ORC:origin complex^5^	REACTOME	11/41	0.03959
DNA Replication^5^ *	REACTOME	11/41	0.03959
Synthesis of DNA^5^	REACTOME	11/41	0.03959
EPO signaling pathway(JAK2 STAT1 STAT3 STAT5)	INOH	5/9	0.04159
Association of licensing factors with the pre-replicative complex^5^	REACTOME	11/42	0.04682

* Interaction Node;

1 S-Phase Network;

2 p53-Independent DNA Damage Response Network;

3 Host interactions of HIV factors Network;

4 Signaling by Wnt Network;

5 DNA Replication.

**Table 6:** Innate DB pathways down-regulated by ES cell derived DCs at 4 hours post-infection.

GO term name of pathways down-regulated at 4 hours after infection	Source Name	GO term down-reg./uploaded genes	GO term down-reg. p-value (corrected)
Citric acid cycle (TCA cycle)^1^	REACTOME	26/103	0.008018
Cori Cycle (interconversion of glucose and lactate)^1^	REACTOME	26/101	0.008018
Glucose metabolism^2^	REACTOME	26/28	0.008018
Oxidative decarboxylation of alpha-ketoglutarate to succinyl CoA by alpha-ketoglutarate dehydrogenase^1^	REACTOME	26/100	0.008018
Pyruvate metabolism and TCA cycle^1^ *	REACTOME	26/96	0.008018
ChREBP activates metabolic gene expression^5^	REACTOME	29/101	0.008137
Integration of energy metabolism^5^ *	REACTOME	29/97	0.008137
PP2A-mediated dephosphorylation of key metabolic factors^5^	REACTOME	29/103	0.008137
Electron Transport Chain^4^	REACTOME	27/94	0.008420
Lysine catabolism^3^	REACTOME	27/95	0.008420
Oxidative decarboxylation of pyruvate to acetyl CoA by pyruvate dehydrogenase^1^	REACTOME	27/95	0.008420
Propionyl-CoA catabolism^6^	REACTOME	27/95	0.008420
Regulation of pyruvate dehydrogenase complex (PDC)^1^	REACTOME	27/95	0.008420
Insulin effects increased synthesis of Xylulose-5-Phosphate^5^	REACTOME	29/95	0.008645
Oxidative decarboxylation of alpha-keto-beta-methylvalerate to alpha-methylbutyryl-CoA by branched-chain alpha-ketoacid dehydrogenase^1^	REACTOME	29/106	0.008740
Gamma-Hexachlorocyclohexane degradation	KEGG	7/106	0.008914
Phosphoenolpyruvate and ADP react to form pyruvate and ATP^2^	REACTOME	28/106	0.009170
Oxidative decarboxylation of alpha-ketoisovalerate to isobutyryl-CoA by branched-chain alpha-ketoacid dehydrogenase^1^	REACTOME	30/92	0.009263
Isoleucine catabolism^3^	REACTOME	29/92	0.009771
Mitochondrial fatty acid beta-oxidation of unsaturated fatty acids^6^ *	REACTOME	31/92	0.009828
Beta oxidation of decanoyl-CoA to octanoyl-CoA-CoA^6^	REACTOME	23/92	0.010158
Beta oxidation of octanoyl-CoA to hexanoyl-CoA^6^	REACTOME	23/92	0.010158
Transcriptional activation of glucose metabolism genes by ChREBP:MLX^5^	REACTOME	28/108	0.010431
Valine, leucine and isoleucine degradation^3^	KEGG	13/11	0.010588
Valine catabolism^3^	REACTOME	29/79	0.010676
Oxidative decarboxylation of alpha-ketoadipate to glutaryl CoA by alpha-ketoglutarate dehydrogenase^1^	REACTOME	27/79	0.010708
Beta oxidation of lauroyl-CoA to decanoyl-CoA-CoA^6^	REACTOME	23/80	0.011196
Beta oxidation of myristoyl-CoA to lauroyl-CoA^6^	REACTOME	23/80	0.011196
Beta oxidation of palmitoyl-CoA to myristoyl-CoA^6^	REACTOME	23/80	0.011196
Beta oxidation of butanoyl-CoA to acetyl-CoA^6^	REACTOME	22/87	0.015703
Oxidative phosphorylation	KEGG	24/77	0.015859
Beta oxidation of hexanoyl-CoA to butanoyl-CoA^6^	REACTOME	22/78	0.018565
**Mets affect on macrophage differentiation** *	PID BIOCARTA	7/13	0.025547
Limonene and pinene degradation	KEGG	7/13	0.025547
Fructose catabolism^2^	REACTOME	33/7	0.027501
**Bisphenol A degradation** *	KEGG	5/140	0.027860
Alanine metabolism^3^	REACTOME	32/135	0.028981
Pentose phosphate pathway (hexose monophosphate shunt)^2^	REACTOME	32/137	0.036902
Dihydroxyacetone phosphate is isomerized to form glyceraldehyde-3-phosphate^2^	REACTOME	32/139	0.045444
Glycolysis^2^	REACTOME	32/139	0.045444

Main interaction nodes were correlated to the data reported by a manual curation in the REACTOME website.

* Interaction Nodes;

1 Pyruvate metabolism and TCA cycle;

2 Metabolism of carbohydrates;

3 Metabolism of amino acids and derivatives;

4 Respiratory electron transport, APT synthesis by chemiosmotic coupling, and heat production by uncoupling proteins;

5 Integration of energy metabolism;

6 Mitochondrial fatty acid beta-oxidation.

The lists of differentially expressed genes obtained by Bioconductor analysis were further analyzed for pathway enrichment analysis using a specialist software package known as InnateDB designed for the focused assessment of immune-associated pathways [24]. The analysis was performed on corresponding human orthologue genes with the fold expression and the p-value reported by Bioconductor analysis at 2 and 4 hours post-infection. This analysis revealed that at 2 hours post-infection, pathways that were significantly differentially expressed (data not shown) included those termed as ‘cytokine-cytokine receptor interaction’, ‘MAPK signaling pathway’, ‘Jak-STAT signaling pathway’, ‘IL-12 mediated signaling events’ and ‘hematopoietic cell lineage’. These pathways have been previously associated with the early response to pathogens [32], [33]. The InnateDB analysis did not reveal any statistically significant down-regulated pathways at 2 hours post infection. Pathway analysis of the genome expression profile at 4 hours compared to 2 hours post-infection reported ‘arachidonic acid metabolism’ as being down regulated. However, the InnateDB analysis of expression profile at 4 hours post-infection comparing *S.* Typhimurium SL1344(p1C/1) versus uninfected ESC derived DCs revealed a number of noteworthy pathways with 18 pathways up-regulated (Table 5) and 40 pathways down-regulated (Table 6). The analysis reported several pathways that are part of the same Network, for example ‘Carbohydrate Metabolism’, ‘DNA-replication’ and ‘Metabolism of amino acids and derivatives’. Up regulated pathways included those termed ‘cytokine-cytokine receptor interaction’, ‘IL-4’, ‘IL-23’ and ‘IL-12 mediated signaling events’. The activation of these particular pathways confirms the immune signature associated with these differentiated ESCs. In particular the identification of the IL-12 pathway suggests that this cytokine although not detected by quantification methods is nevertheless expressed. It is possible that this protein is expressed in low concentration not detectable by our assays. IL-23 is part of the IL-12 family and shares the subunit p40 with IL12 itself [34]. These two cytokines can have a proinflammatory effect connecting innate and adaptive immune responses via the activation and differentiation of T cells [35], [36]. Patients with inherited deficiency of these cytokines are more susceptible to salmonellosis [37]. IL-4 pathway, which is used for in vitro differentiation into DCs was also up-regulated during *Salmonella* infection [38], [39]. Other interesting pathways include the ‘Vpu mediated degradation of CD4’. Vpu is an HIV protein that, on association with CD4, induces self degradation by the proteasome. It is possible that *Salmonella* induce CD4 degradation in a similar way as suggested by the observation of the lack of CD4 positive DCs at 5 days post-*Salmonella* infection in B cell follicle borders in the spleen [40]. Among the down-regulated pathway at 4 hours post-infection reported by the InnateDB analysis, many can be connected to a few network (Table 6) including ‘pyruvate metabolism and TCA cycle’, ‘Energy metabolism’, ‘Fatty acid β-oxidation’. These pathways are all involved in the mitochondrial metabolism and *Salmonella* is known to target this organelle with specific effector proteins [41]. The variety of Interaction Nodes reported to be up-regulated in our analysis highlight the deep remodeling process that the differentiated cells undergo during bacterial invasion in contrast to undifferentiated ESCs.

## Discussion

We showed previously that undifferentiated ESCs can be infected with bacterial pathogens including *S.* Typhimurium and *S. flexneri* [16]. However, such infected cells exhibited no obvious immune signature and consequently they are not ideal to explore immune responsiveness to invading bacteria. DCs are specialized sentinel cells of the immune system, able to detect the presence of pathogens. Therefore, here ESCs were differentiated along this lineage and the impact of exposure to the invasive *S.* Typhimurium SL1344(p1C/1), which is known to target and exploit such cells, was investigated. We showed through a combination of flow cytometric analysis, microscopy and transcriptome profile that the ESDCs respond robustly to exposure to *S.* Typhimurium SL1344 and associated stimuli such as LPS or TNFα. They were able to express immune surface markers, secrete cytokines and even present MHC class II antigen to activate T cells hybridoma cell line in an *in vitro* assay. These ESDCs were CD8^−^ and expressed markers such as CD4, CD11b, and CD11c. Based on these characteristics [42], *in vitro* differentiated ESDCs resemble *in vivo* DCs present in the spleen and in the intestinal Peyer’s patches. Evidence from transcriptomic and cytokine analysis indicates that an array of different immune pathways was activated by exposure to pathogenic insult by ESDCs. Interestingly, we were unable to quantify IL-12 protein even though evidence from the transcriptome analysis suggested the gene is activated during infection. It is possible that deriving from embryonic cells, ESDCs may be more ‘primitive’ or less differentiated than similar cells developing *in vivo* during hematopoiesis. Nevertheless the potential of this model is evident and in the future it may be possible to optimize protocols to obtained specific subsets of specialized DC from murine and perhaps human induced pluripotent stem cells (iPSC) opening the opportunity to investigate the interaction of pathogenic bacteria with human DCs [43]. These approaches may also find utility in the exploitation of mutant mouse and human ESC lines [44], [45], [46]. In conclusion, we believe that this stem cell based approach has a great potential in the future studies of host-pathogen interactions.
